# Estimating the burden of influenza‐associated hospitalizations and deaths in Chile during 2012‐2014

**DOI:** 10.1111/irv.12502

**Published:** 2018-02-14

**Authors:** Viviana Sotomayor, Rodrigo A. Fasce, Natalia Vergara, Felipe De la Fuente, Sergio Loayza, Rakhee Palekar

**Affiliations:** ^1^ Department of Epidemiology Ministry of Health of Chile Santiago Chile; ^2^ Sub‐Department of Viral Diseases Instituto de Salud Pública de Chile Santiago Chile; ^3^ Pan‐American Health Organization Washington D.C. USA

**Keywords:** burden of disease, Chile, hospitalizations, influenza, mortalitiy

## Abstract

**Background:**

Influenza is a vaccine preventable disease that causes important morbidity and mortality worldwide. Estimating the burden of influenza disease is difficult. However, there are some methods based in surveillance data and laboratory testing that can be used for this purpose.

**Objectives:**

Estimating the burden of serious illness from influenza by means of hospitalization and death records during the period between 2012 and 2014, and using information from Severe Acute Respiratory Illness (SARI) surveillance.

**Methods:**

To estimate the Chilean rate of influenza‐associated hospitalizations and deaths, we applied the influenza positivity of respiratory samples tested in six SARI surveillance sentinel hospitals to the hospitalizations and deaths from the records with ICD‐10 codes from influenza and pneumonia.

**Results:**

Annually, 5320 people are hospitalized for influenza and 447 die for this cause. The annual influenza‐associated hospitalization rate for the period was 71.5/100 000 person‐year for <5 years old, 11.8/100 000 person‐year for people between 5 and 64 years old; and 156.0/100 000 person‐year for ≥65 years. The annual mortality rate for the period was 0.08/100 000 person‐year for <5 years; 0.3/100 000 person‐year for people between 5 and 64 years; and 22.8/100 000 person‐year for ≥65 years.

**Conclusions:**

This is the first study of influenza burden in Chile. Every year an important quantity of hospitalizations and deaths result from influenza infection. In countries in temperate zones, it is important to know the burden of influenza in order to prepare the health care network and to assess preventive intervention currently in practice and the new ones to implementing.

## 1 INTRODUCTION

Influenza is a vaccine‐preventable disease that occurs worldwide and causes important morbidity and mortality, especially in high‐risk groups such as children, adults 65 years of age and older, pregnant women, and individuals with chronic diseases. Transmission of influenza tends to increase during the winter season in countries with temperate climates, often severely impacting the healthcare services network.^1^ The Ministry of Health (MoH) of Chile (MINSAL), before the seasonal rise and prior to the seasonal increase in influenza transmission, carries out annual vaccination campaigns against influenza, following the World Health Organization (WHO) recommendation for the composition of the Southern Hemisphere influenza vaccine. The target groups for vaccination have been children between 6 to 24 months of age and 2 to 5 years of age (since 2015), as well as adults 65 years of age and older, pregnant women, chronically ill persons, and the healthcare personnel.^2^, ^3^, ^4^ In Chile, target groups for vaccination were incorporated early into the country's national policies.^5^

As awareness of the risks associated with influenza infection and the importance of influenza monitoring has increased, the surveillance system for influenza and other respiratory viruses has been strengthened in Chile. The system is currently based on sentinel surveillance for influenza‐like illness (ILI), severe acute respiratory infection (SARI), and monitoring of visits to emergency wards; additionally, a decentralized laboratory network plus the *Instituto de Salud Pública* of Chile (ISP), which is a National Influenza Center recognized by WHO,^6^ conduct virologic surveillance of influenza. The sentinel influenza‐proxy syndromic surveillance for SARI has been progressively implemented in the country since 2011 with the aim of characterizing severe acute respiratory infections associated with influenza and of monitoring the severity of influenza virus subtypes.^7^ However, the burden of influenza disease is difficult to estimate, because influenza virus produces a wide range of symptoms and syndromes not very specific and it is unlikely that laboratory confirmation of cases is carried out routinely.^8^, ^9^ Additionally, the seasonal increase in influenza virus coincides with that of other respiratory viruses that can cause a similar clinical picture and often bacterial superinfection occurs. This situation, in addition to making it difficult to measure the burden of disease caused by influenza, hinders the estimation of the impact of existing prevention and treatment measures, as well as, the analysis of health interventions.^8^, ^9^

Population‐based cohort studies likely provide the most accurate direct measurement of ambulatory influenza disease burden; however, cohorts are not practical to measure hospitalized influenza burden and are extremely costly. There are some strategies that allow for the estimation of influenza burden of disease using data from epidemiological surveillance when it is associated with the use of laboratory techniques for confirmation. In the Americas, several estimation analyses have been carried out using these methods, mainly in the United States, but also in Central America and the Caribbean.^10^, ^11^, ^12^, ^13^, ^14^, ^15^, ^16^, ^17^ In Chile, despite having a stable surveillance system with quality data, to date, no burden estimates have been made using these strategies.

Thus, the objective of this analysis was to estimate the burden of serious illness from influenza through analysis of hospitalization and death records for the period during 2012‐2014 and laboratory surveillance among SARI cases in Chile. These estimates will allow national authorities to have relevant information for the impact of prevention and control strategies.

## 2 METHODS

To estimate the burden of influenza‐associated hospitalizations and deaths in Chile, a WHO published methodology was used.^12^, ^18^ Virologic surveillance data and the number of respiratory hospitalizations and deaths, at national level, were paired to estimate the amount of influenza circulating. Representativeness of virologic and SARI sentinel surveillance was assumed.

### 2.1 Information sources

The following data were used to perform this analysis:

1. Records of hospital discharges, which are managed by the Department of Statistics and Health Information (DSHI) of the MoH of Chile,^19^ were obtained. These records include all public and military hospitals and over 90% of the private hospitals within the 15 regions of the country.
2. Records of national deaths, managed by DSHI, were obtained. These records contain information on more than 99% of the deaths occurred in the country, with a percentage of ill‐defined from 2.2% to 2.6%^20^ during 2012‐2014.
3. The population database from 2002 to 2012 and population projections from 2013, performed by the National Institute of Statistics,^21^ were obtained. For calculation of average rates, during 2012‐2014, the population during 2013 was used.
4. The SARI sentinel surveillance database^22^ that contains the records from the six SARI sentinel sites was obtained. These sites are distributed across three of the four geographic‐administrative macro zones of the country (north, center, and center south), where more than 80% of the population lives. The personnel in charge of surveillance is trained annually in SARI case identification definition using the following case definition—a patient who is hospitalized with fever or history of fever of 38°C or higher, cough, and respiratory distress (ie, polypnea and decreased oxygen saturation).^7^ For patients meeting the case definition, a standard form is completed that includes demographic information, date of onset of symptoms, date of clinical sample collection and sample testing, influenza vaccination history, presence of risk factors for adverse outcome, and history of use of the healthcare system. A nasopharyngeal aspirate or a nasopharyngeal swab is also collected, as per the ISP laboratory protocol.^23^ All clinical samples collected from SARI cases are tested by immunofluorescence (IF) for a panel of respiratory viruses (RSV, parainfluenza, adenovirus, influenza A, influenza B, and human metapneumovirus). The cases that test negative by IF or positive for influenza are further tested by real‐time RT‐PCR for influenza A and influenza B. All influenza A cases are then subtyped by real‐time RT‐PCR for H3 and H1pdm09^7^ subtypes. During the study period, samples were collected from 98.0% of the SARI cases and all of the IF‐negative cases were tested by real‐time RT‐PCR.

### 2.2 Estimation of the rate of hospitalizations and deaths associated with influenza by age‐group

For the analysis, the counts of the number of persons hospitalized or deceased (obtained from records of national hospitalizations and deaths) due to influenza or pneumonia (International Classification of Disease [ICD] 10 codes J09 to J18) as a primary or basic cause of death, for the period between January 1, 2012 to December 31, 2014, were included. ICD 10 codes were chosen to compare our result with previous studies from the USA and Central America and Caribbean. The census population was stratified into three age‐groups: <5 years old, 5‐64 years of age, and ≥65 years of age and older.

The rate of influenza‐associated hospitalizations was calculated applying the percentage of monthly samples positive for influenza obtained from SARI surveillance to the monthly number of hospital discharges due to influenza and pneumonia (J09‐J18) at the national level. For the estimation of influenza‐associated deaths, the same monthly influenza positivity obtained from SARI surveillance was used and applied to the monthly number of mortality records for pneumonia and influenza. These monthly counts were summed to estimate a total number of influenza‐associated hospitalizations or deaths for the specified year. To determine the denominator of the calculation of the annual hospital and death rate, it was assumed that each inhabitant had the same risk of developing a serious illness by influenza and of being hospitalized and as such the census population for the country was used as the denominator.

The hospitalization rate calculation is summarized by the following formula:

$$I_{i}=\frac{\sum_{m}E_{i,m}\times \frac{P_{i,m}}{T_{i,m}}}{B_{i}}$$

where: *I*_*i*_: hospital incidence associated with influenza for each age‐group; *E*_*i,m*_: number of hospital discharges with J09‐J18 diagnostics, for each age‐group and month; *P*_*i,m*_: number of SARI cases with influenza‐positive samples, for each age‐group and month; *T*_*i,m*_: total number of SARI cases with samples collected, for each group and month; *B*_*i*_: census population for each age group; *i*: age‐group (<5, 5‐64 and ≥65 years); *m*: month.

The same formula was used for mortality, replacing *E*_*i*,*m*_, for the number of deaths each month.

Calculation of 95% confidence interval (CI) is defined as:

$${\text{IC}}_{95\%}\left(I_{i}\right)=\left[\frac{I_{i}}{\text{EF}};I_{i}\times \text{EF}\right]$$

where: $\text{EF}=e^{\left(1.96\times \sqrt{d}\right)}$ *, d* corresponds to the number of influenza cases.

The rate of hospitalizations and deaths associated with influenza were estimated for each year and for the period 2012‐2014. In the latter case, the mean of annual incident cases was used and the number of people in the middle of the period was considered as the population at risk.

The median of positivity and its interquartile range (IQR) for each year and for the whole period was also calculated. The analysis was also performed by age‐group and by viral type and subtype for influenza.

Microsoft Excel 2013 and stata 12.1 (StataCorp, Lakeway Drive, TX, USA), were used for all calculations.

The analysis was carried out using anonymized databases under the epidemiological surveillance model, respecting the national legislation on the management of clinical data and confidentiality, and therefore did not require approval of an Ethics Committee.

## 3 RESULTS

A total of 185 379 hospitalizations with diagnosis of pneumonia or influenza were registered in Chile during 2012‐2014; of these, 34.9% were children younger than 5 years of age, 23.6% were people between 5 and 64 years of age, and 41.5% were people older than 65 years of age (Table 1).

**Table 1:** Influenza‐associated SARI hospitalizations and deaths in Chile, 2012‐2014

Year and age‐group	Population size	No. of hospitalization for influenza and pneumonia^a^	No. of deaths for influenza and pneumonia^b^	Positivity (%)^c^ (No. of samples tested)	Influenza‐associated hospitalizations		Influenza‐associated death	
					Number	Rate^d^ (95% CI)	Number	Rate^d^ (95% CI)
2012								
<5 y	1 238 021	24 025	25	3.5 (1.823)	1027	82.9 (78.06‐88.21)	1	0.08 (0‐0.5)
5‐64 y	14 544 736	14 772	364	13.3 (706)	1613	11.9 (10.5‐11.6)	43	0.29 (0.2‐0.3)
≥65 y	1 662 042	27 970	3466	15.6 (717)	3366	202.5 (195.8‐209.5)	427	25.71 (23.3‐28.2)
2013								
<5 y	1 239 740	21 421	26	5.2 (1.957)	1078	86.92 (81.8‐92.2)	1	0.08 (0‐0.5)
5‐64 y	14 667 477	16 066	442	23.5 (1.086)	2653	18.09 (17.4‐18.7)	80	0.54 (0.4‐0.6)
≥65 y	1 724 362	25 340	3906	12.1 (889)	2370	135.31 (129.9‐140.9)	370	21.43 (19.3‐23.7)
2014								
<5 y	1 238 097	19 308	24	3.0 (2.358)	557	45 (41.4‐48.8)	1	0.07 (0‐0.5)
5‐64 y	14 791 488	12 927	462	8.5 (1.227)	963	6.5 (6.1‐6.9)	34	0.22 (0.1‐0.3)
≥65 y	1 789 469	23 550	3791	12 (1.065)	2368	132.48 (127.2‐137.9)	386	21.55 (19.5‐23.8)
Media 2012‐2014								
<5 y	1 239 740	64 754	75	3.8 (6.138)	887	71.5 (67‐76.4)	1	0.08 (0‐0.5)
5‐64 y	14 667 477	43 765	1268	15.0 (3.019)	1743	11.8 (11.3‐12.4)	52	0.3 (0.2‐0.4)
≥65 y	1 724 362	76 860	11 163	13.2 (2.671)	2690	156.0 (150.2‐162)	394	22.8 20.7‐25.2)

SARI, severe acute respiratory illness.

a Number of persons hospitalized during 2012‐2014 for SARI proxy diagnoses (ICD‐10 code J09‐J18).

b Number of persons death during 2012‐2014 for SARI proxy diagnoses (ICD‐10 code J09‐J18).

c Percentage of respiratory sample positive for influenza by immunofluorescence and PCR over the total tested.

d Per 100 000 person‐year.

During the same period, 11 828 SARI cases were reported and tested for influenza from the six surveillance sentinel hospitals; of these, 51.9% were children younger than 5 years of age, 25.5% were persons aged between 5 and 64 years of age, and 22.6% were 65 years of age and older. The overall positivity for influenza was 3.8% among children <5 years of age, 15.0% among those 5‐64 years of age, and 13.2% among those 65 years of age and older. Table 2 shows the annual influenza positivity for each group of age with the monthly median and IQR.

**Table 2:** Positivity for influenza of respiratory samples in SARI sentinel surveillance. Chile 2012‐2014

Year and age‐group	No. of samples tested	Positivity (%)^a^	Median of monthly positivity, % (Interquartile range, %)
2012			
Total	3246	8.3	7.0 (2.3‐8.4)
<5 y	1823	3.5	2.9 (0.4‐5.2)
5‐64 y	706	13.3	8.4 (1.7‐12.3)
≥65 y	717	15.6	8.4 (0.0‐14.3)
2013			
Total	3932	11.8	4.8 (3.7‐7.6)
<5 y	1957	5.2	3.1 (1.8‐4.9)
5‐64 y	1086	23.5	7.4 (4.7‐14.4)
≥65 y	889	12.1	6.9 (4‐10.5)
2014			
Total	4650	6.6	4 (3.0‐6.7)
<5 y	2358	3.0	1.8 (1.1‐4.1)
5‐64 y	1227	8.5	6.4 (3.2‐9.4)
≥65 y	1065	12.4	7.4 (3.4‐12.7)
Average 2012‐2014			
Total	11 828	8.8	4.8 (2.9‐8.3)
<5 y	6138	3.8	2.5 (1.2‐4.7)
5‐64 y	3019	15.0	7.6 (4.1‐11.4)
≥65 y	2671	13.2	6.9 (3.3‐13.9)

SARI, severe acute respiratory illness.

a Percentage of respiratory sample positive for influenza by immunofluorescence or PCR over the total tested.

**Figure 1:**
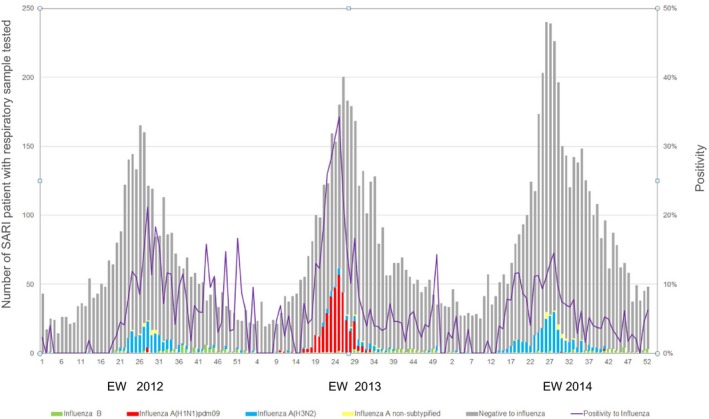
Number of severe acute respiratory illness patient with respiratory samples tested and positivity to influenza in six SARI sentinel hospitals. Chile 2012–2014

Among the SARI clinical samples, influenza A predominated over influenza B (75.6%, 88.1%, and 84.6% for 2012, 2013, and 2014, respectively). The predominant influenza A subtype during 2012 and 2014 was influenza A(H3N2) (74.0% and 82.9%, respectively) and influenza A(H1N1)pdm09 during 2013 (74.6%) (Figure 1).

The average number of annual influenza‐associated hospitalizations in Chile was 887 (16.7%) in children younger than 5 years of age, 1743 (32.8%) in people between 5 and 64 years, and 2690 (50.2%) in adults ≥65 years. The average annual rate of hospitalizations was 71.5 (95% CI: 67.0‐76.4), 11.8 (95% CI: 11.3‐12.4), and 156.0 (95% CI: 150.2‐162.0) per 100 000 person‐years, respectively. With regard to the group aged between 5 and 64 years old, the risk of influenza‐associated hospitalization was 6.1 times as high as that among children <5 years of age and 13.2 times as high as that among those 65 years of age and older (Table 1).

Influenza A‐associated hospitalizations were predominant during the period, but the importance of those related to influenza B increased when compared with the type distribution in SARI samples, reaching a 33.9% in 2012 and approximately a 21% in 2013 and 2104 (Table 3).

**Table 3:** Distribution by type and subtype of positive respiratory samples to influenza in SARI hospitalizations and influenza‐associated hospitalizations. Chile 2012‐2014

Years	SARI hospitalizations tested (%)			Influenza‐associated hospitalizations (%)		
	Influenza A(H1N1)pdm09	Influenza A(H3N2)	Influenza B	Influenza A(H1N1)pdm09	Influenza A(H3N2)	Influenza B
2012	1.6	74.0	24.4	1.8	64.3	33.9
2013	74.6	13.5	11.9	58.8	20.0	21.2
2014	0.7	82.9	16.4	1.1	78.3	20.6

SARI, severe acute respiratory illness.

During the analyzed period, we identified 12 506 deaths due to influenza and pneumonia: 0.6% (75) in children less than 5 years of age, 10.1% (1268) in the those aged 5‐64 years of age, and 89.3% (11 163) among those 65 years of age and older.

The average of annual number of influenza‐associated deaths in Chile was one in children less than 5 years of age, 52 among persons 5‐64 years of age, and 394 among adults 65 years of age and older. The annual mortality rate was 0.08 (95% CI: 0‐0.5), 0.3 (95% CI: 0.2‐0.4), and 22.8 (95% CI: 20.7‐25.2) per 100 000 person‐years, respectively. The mortality rate in the group aged 65 years and older was 76 as high as and 285 times as high as the risk in the group of 5 and 64 years and children <5 years, respectively. For each age‐group, there was no statistically significant difference in the influenza‐associated mortality rate, with the exception of 2013 in the group 5‐64 years of age, when the mortality rate (0.54 person‐year per 100 000) was almost twice as high as the rates during 2012 and 2014.

Overall, the risk by age was similar between type and subtypes of influenza virus; however, there were some small differences observed. The risk of hospitalization for the group of 5‐64 years of age was higher with influenza A(H1N1)pdm09 than influenza A(H3N2) when comparing years and the relation with the two another age‐groups. Otherwise, the difference in risk between age‐groups was smaller. Table 4 shows the rates and distribution of the influenza‐associated hospitalizations by type and subtype year and age‐group.

**Table 4:** Rate by type and subtype of influenza‐associated hospitalizations. Chile 2012‐2014

Year and Age‐group	A(H1N1)pdm09 influenza‐associated hospitalizations			A(H3N2) influenza‐associated hospitalizations			Influenza B‐associated hospitalizations.		
	Positivity (%)^a^	Influenza cases	Rate^b^ (95% CI)	Positivity (%)^a^	Influenza cases	Rate^b^ (95% CI)	Positivity (%)^a^	Influenza cases	Rate^b^ (95% CI)
2012									
<5 y	0.05	15	1.2 (0.7‐2)	2.4	708	57.1 (53.1‐61.5)	0.8	213	17.1 (15‐19.6)
5‐64 y	0.0	26	0.1 (0.1‐0.2)	8.9	297	2 (1.8‐2.2)	3.1	552	3.7 (3.4‐4.1)
≥65 y	0.3	49	2.9 (2.2‐3.9)	11.3	2242	134.9 (129.4‐140.6)	3.6	950	57.1 (53.6‐60.8)
2013									
<5 y	3.4	502	40.4 (37‐44.1)	0.6	118	9.5 (7.9‐11.3)	0.8	132	10.6 (8.9‐12.6)
5‐64 y	19.5	1761	12 (11.4‐12.5)	1.6	204	1.3 (1.2‐1.5)	1.9	467	3.1 (2.9‐3.4)
≥65 y	6.6	964	55.8 (52.4‐59.5)	3.6	776	44.9 (41.9‐48.2)	2.0	562	32.6 (30‐35.4)
2014									
<5 y	0.0	0	0	2.1	401	32.4 (29.3‐35.7)	0.4	79	6.3 (5.1‐7.9)
5‐64 y	0.0	0	0	7.1	759	5.1 (4.7‐5.5)	1.5	220	1.4 (1.3‐1.7)
≥65 y	0.2	44	2.4 (1.8‐3.2)	10.3	1836	102.6 (98‐107.4)	1.9	488	27.2 (24.9‐29.8)

CI, confidence interval.

a Percentage of respiratory sample positive for influenza by immunofluorescence and PCR over the total tested.

b Per 100 000 persons‐year.

## 4 DISCUSSION

This is the first analysis to estimate the burden of severe influenza in Chile. An important number of people throughout all ages are hospitalized for influenza annually; however, the risk of serious influenza illness was greatest in the extreme ages, being about six times as high as and 13 times as high as among children younger than 5 years of age and the elderly of 65 and more years old, as compared to people between 5 and 64 years of age.

As influenza is known to cause more severe disease among the age extremes as compared to persons aged 5‐64 years of age, our finding is consistent with previously published findings (CDC). Furthermore, a higher percent positivity for influenza was reported among the older age‐group as compared to the younger age‐groups, which provides further evidence of the greater burden in this age‐group. This pattern is maintained for all viral subtypes; however, the differences tend to decrease with influenza A(H1N1) perhaps due to the published finding that influenza(H1N1)pdm09 affects younger adults more than older ones due to absence of immune‐protection against this virus (USA, Mexico, Brazil, four European countries).^24^, ^25^, ^26^

Our estimates for persons aged 65 years and older are greater than those reported in an analysis from Central America using the same data source (ie, pneumonia and influenza codes) and rate calculation methodology (44 per 100 000 persons‐year).^12^ This might be due to differences in the ascertainment of cases that are tested for influenza or completeness of the hospital discharge and death databases. Also, this could be related to other aspects such as vaccination coverage, practices of hospitalization, and health‐seeking behavior that could be different in this subregion of the Americas as compared to Chile. On the other hand, our estimates in this group are similar to the estimates from the United States (170‐1033 influenza‐associated hospitalizations per 100 000 persons‐years).^17^

Our estimates found among children under 5 years of age are similar to the global meta‐analysis for countries in the Americas (71 influenza‐associated hospitalizations per 100 000 persons‐years)^27^ and lower than the estimates from Central American countries (113 influenza‐associated hospitalization per 100 000 persons‐year).^12^ Again this is likely attributable to various country‐level behaviors and surveillance system characteristics as explained above.

Overall, our under‐5 years of age mortality estimates are low and could be explained by the countrywide respiratory diseases management and prevention strategy that is applied during the winter influenza season that focuses upon interventions in this risk group, such as preventing and treating early complications in of illness among children, whether due to influenza or other respiratory viruses. This lower rate could also be explained by the coverage of vaccination in children from 6 to 24 months of age, which on average in the period 2012‐2014 was 91%.^28^

In our study, the estimated mortality rates are only slightly lower than those observed in the meta‐analysis of the Central American countries (1.3 cases per 100 000 persons‐years)^12^ and of other global studies (4.9 cases per 100 000 habitants,^29^ for developing countries). In the elderly, our estimated mortality rates are higher than those reported by other studies in Central America (7.3 cases per 100 000 inhabitants).^12^

Rates varied by subtype, being higher in the elderly when the predominant circulating influenza subtype is influenza A(H3N2). The years in which this subtype predominated have been associated with an increase in hospitalizations and deaths in both the elderly and children under 5 years of age in relation to the years in which other subtypes predominated.^30^, ^31^

This analysis is subject to several limitations. First, we used a proxy to quantify the number of severe influenza cases—the hospital discharges or deaths due to pneumonia and influenza at the national level, and the application of the surveillance percent positivity for influenza from six hospitals to these cases, which might under‐estimate the mortality rates, especially in the extreme age‐groups, where the suspicion of influenza is low in comparison with other etiologies for the death. Also, analyzing exclusively hospital discharges due to pneumonia and influenza, excluding those cases that had chronic diseases in which the influenza infection triggered the hospitalization, or those cases where the clinical pattern is not a typical flu or pneumonia. Further analysis must be performed with a more sensitive definition like ICD 10 J00 to J99. Next, the study period is too short to account for the variability between influenza seasons and viral subtypes. Finally, although the hospitals that perform SARI surveillance apply the same protocols for capturing cases, there may be variations that are related to the intensity to maintain and prioritize sampling throughout the year, especially in adults.

## 5 CONCLUSION

Annually, between 4000 and 6500 hospitalizations associated with influenza are expected at the national level in Chile, with the highest risk group being those over 65 years of age followed by those under 5 years of age. In relation to mortality, between 450 and 500 deaths are expected to be associated with influenza in Chile, being mostly among those 65 years of age and older.

This analysis highlights the importance of maintaining risk‐based prevention and control strategies including the use of the influenza vaccine; further analyses should assess vaccine impact as well as estimate economic burden and medical burden during more seasons. Additionally, resources should continue to be devoted to maintain SARI surveillance, which allows the monitoring of patterns of severe influenza disease.
